# Research on Emissions, Air quality, Climate, and Cooking Technologies in Northern Ghana (REACCTING): study rationale and protocol

**DOI:** 10.1186/s12889-015-1414-1

**Published:** 2015-02-12

**Authors:** Katherine L Dickinson, Ernest Kanyomse, Ricardo Piedrahita, Evan Coffey, Isaac J Rivera, James Adoctor, Rex Alirigia, Didier Muvandimwe, MacKenzie Dove, Vanja Dukic, Mary H Hayden, David Diaz-Sanchez, Adoctor Victor Abisiba, Dominic Anaseba, Yolanda Hagar, Nicholas Masson, Andrew Monaghan, Atsu Titiati, Daniel F Steinhoff, Yueh-Ya Hsu, Rachael Kaspar, Bre’Anna Brooks, Abraham Hodgson, Michael Hannigan, Abraham Rexford Oduro, Christine Wiedinmyer

**Affiliations:** National Center for Atmospheric Research, PO Box 3000, Boulder, CO 80307 USA; Navrongo Health Research Centre, Behind Navrongo War Memorial Hospital, Navrongo, Ghana; University of Colorado – Boulder, Boulder, CO 80309-0427 USA; Relief International, 5455 Wilshire Blvd., Suite 1280, Los Angeles, CA 90036 USA; EPA Human Studies Facility, 104 Mason Farm Road, Chapel Hill, NC 27514-4512 USA; Ghana Health Service, Private Mail Bag, Ministries, Accra, Ghana

**Keywords:** Cookstoves, Household air pollution, Global health, Study protocol, Randomized intervention study

## Abstract

**Background:**

Cooking over open fires using solid fuels is both common practice throughout much of the world and widely recognized to contribute to human health, environmental, and social problems. The public health burden of household air pollution includes an estimated four million premature deaths each year. To be effective and generate useful insight into potential solutions, cookstove intervention studies must select cooking technologies that are appropriate for local socioeconomic conditions and cooking culture, and include interdisciplinary measurement strategies along a continuum of outcomes.

**Methods/Design:**

REACCTING (Research on Emissions, Air quality, Climate, and Cooking Technologies in Northern Ghana) is an ongoing interdisciplinary randomized cookstove intervention study in the Kassena-Nankana District of Northern Ghana. The study tests two types of biomass burning stoves that have the potential to meet local cooking needs and represent different “rungs” in the cookstove technology ladder: a locally-made low-tech rocket stove and the imported, highly efficient Philips gasifier stove. Intervention households were randomized into four different groups, three of which received different combinations of two improved stoves, while the fourth group serves as a control for the duration of the study. Diverse measurements assess different points along the causal chain linking the intervention to final outcomes of interest. We assess stove use and cooking behavior, cooking emissions, household air pollution and personal exposure, health burden, and local to regional air quality. Integrated analysis and modeling will tackle a range of interdisciplinary science questions, including examining ambient exposures among the regional population, assessing how those exposures might change with different technologies and behaviors, and estimating the comparative impact of local behavior and technological changes versus regional climate variability and change on local air quality and health outcomes.

**Discussion:**

REACCTING is well-poised to generate useful data on the impact of a cookstove intervention on a wide range of outcomes. By comparing different technologies side by side and employing an interdisciplinary approach to study this issue from multiple perspectives, this study may help to inform future efforts to improve health and quality of life for populations currently relying on open fires for their cooking needs.

## Background

Biomass-burning cookstoves are widely recognized as a significant source of pollutants impacting human health, local and regional air quality, and global climate change. Worldwide, it is estimated that three billion people use biomass- and coal-burning fires to cook and heat their homes [1]. This widespread practice contributes to several health, environmental, and social problems. Nearly four million people are estimated to die prematurely each year due to household air pollution from biomass burning [2]. Exposure to household air pollution from burning biomass fuels has been linked to significant morbidity and mortality from acute lower respiratory infections in children [3], as well as chronic obstructive pulmonary disease [4] and increased rates of cardiovascular disease [5,6] among women, along with other health issues [7]. Local environmental impacts include deforestation and land cover change associated with fuelwood collection [8]. Gathering fuel is also a time consuming activity, particular in arid regions such as our study area in Northern Ghana. This burden falls largely on women and children, contributing to “time poverty” [9] that, along with the health burden that falls disproportionately on women (and children – particularly female children), limits opportunities for female empowerment and development. In addition, cookstove emissions of greenhouse gases, particulate black carbon, and other air pollutants contribute to degraded air quality and global climate change (e.g., [10-12]).

Despite growing attention to the wide-ranging negative impacts of cooking with biomass, efforts to better understand and find solutions to this problem have faced a number of common challenges. These challenges include matching stove technologies to local socioeconomic conditions and cooking culture, and designing comprehensive measurement strategies to effectively diagnose reasons for the success or failure of a given intervention along a continuum of steps in the causal chain from a stove intervention to outcomes of interest. Together, these challenges call for integrated, interdisciplinary approaches to the design of cookstove studies and policies.

### Challenges in stove selection and adoption

Technologically, the problem of open fire cooking using solid fuels seems relatively straightforward to address: a wide variety of improved cookstoves and cleaner fuel sources exist that are more efficient and can reduce air pollutant emissions. Yet efforts to make these technologies available in areas of need throughout the world have often failed to achieve their intended results [2,13]. Human behaviors – specifically, acceptance and use of improved stoves – are key to the success of any cookstove intervention [2,13-18].

Two key and related challenges are locally appropriate stove selection and promotion by those introducing new technologies, and sustained stove adoption and use among target populations [19]. By stove selection, we are referring to the processes of selecting the “right” technology (or mix of technologies) that is most likely to meet the needs of the target population while achieving meaningful reductions in negative health and environmental impacts. Some argue that only the cleanest, most advanced, and usually imported cooking technologies should be promoted, since these have the highest probability of having meaningful impacts on health and environmental outcomes. Others contend that introducing affordable, feasible, locally-produced cookstoves that are more efficient than open fires and more aligned with the unique cooking practices and needs of a given context can be an effective first step toward moving households up the “technology ladder” in the long run [20,21].

Conceptually, the stove or energy “ladder” model is rooted in a neo-classical understanding of energy use that implies cleaner fuel usage with rising socioeconomic status [18]. Typically, this model also implicitly assumes that households rely on a single source of cooking energy at any given time. Empirically, however, studies have found that rather than moving linearly up this energy ladder in a step-by-step fashion, households often rely simultaneously on multiple types of fuel and cooking technologies to meet their cooking needs [15,18]. This energy or technology “stacking” allows households greater flexibility: they can use different types of stoves for different purposes, or alternate among different fuels (essentially moving both up and down the ladder) depending on availability and cost [15,18,22-24]. Of course, these two models may both be correct in some respects; while households may continue to use a mix of technologies, it is possible that the technologies that comprise the cooking “stack” may become cleaner over time.

In light of this view of how energy transitions occur, it is perhaps not surprising that many stove intervention studies have observed that households continue to use their traditional stoves alongside improved stoves [14,17,18,22,24]. Furthermore, the extent to which new stoves are folded into the technology stack and can ultimately displace traditional cooking methods (leading to cleaner kitchens overall) depends heavily on how well suited these new technologies are to local culture and cooking practices [15]. For example, a study of cooking practices in Guatemala showed that more affluent households (receiving remittances from migrant family members) had liquid petroleum gas (LPG) stoves but continued to rely on wood-burning stoves for most of their cooking needs because these stoves were better suited to the preparation of staple food items (beans, corn, and tortillas) [17]. Ultimately, without incorporating traditional cooking practices into the design process, even low-cost stoves are unlikely to be used [25].

### Assessing stove intervention success

**Figure 1:**
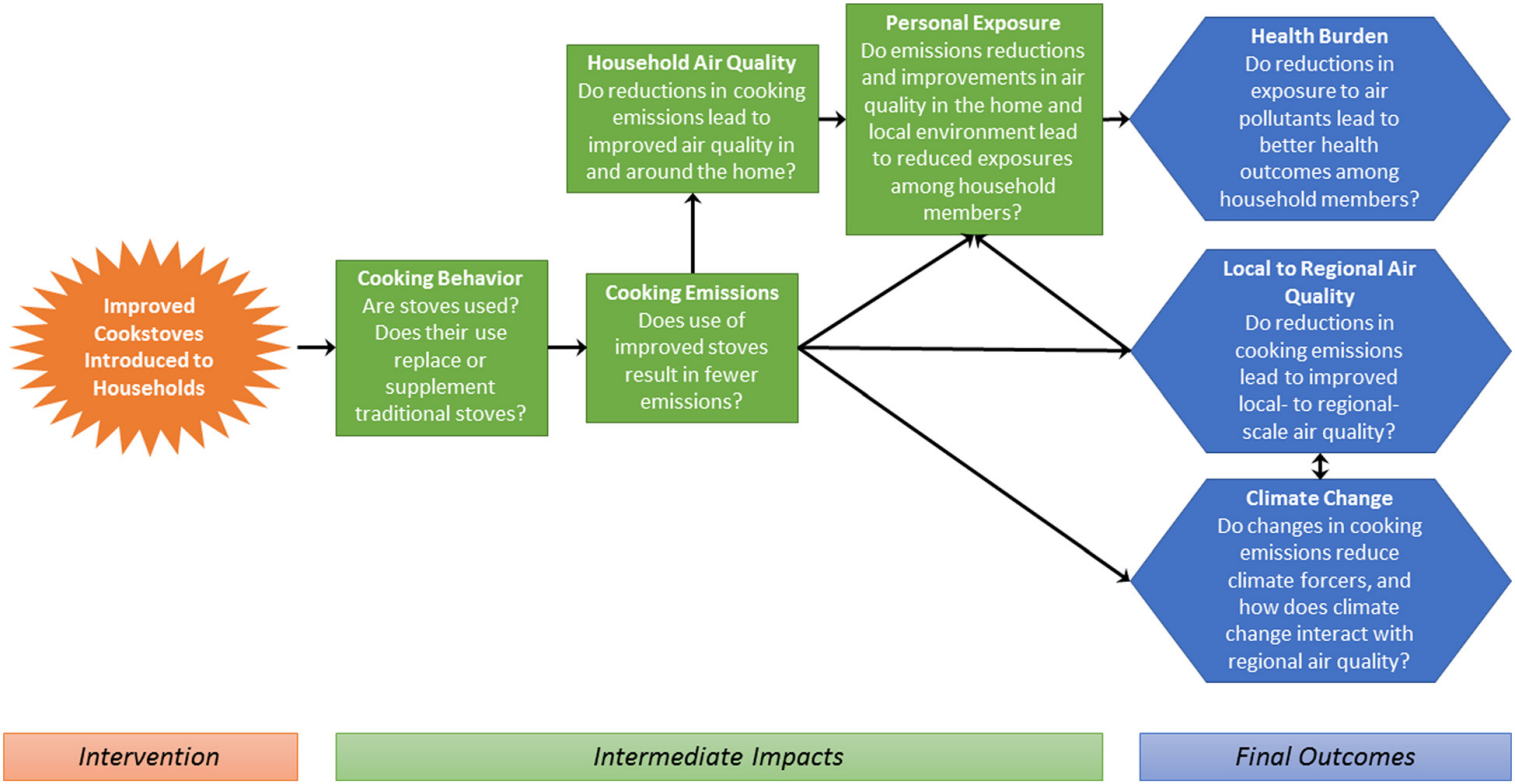
**Causal pathways linking introduction of clean cookstoves to outcomes of interest.**

Stove intervention studies are motivated by the large potential impacts of improved stove use on several final outcomes, from public health to environmental quality. However, there are a number of intermediate steps linking the introduction of a new technology to these final outcomes. The causal chain connecting a stove intervention to three key endpoints, health burden, local to regional air quality, and climate change, is shown in Figure 1. Understanding this causal chain, and where it may break down, is essential to learning about what makes a particular intervention (in)effective, and how future endeavors can improve upon existing efforts.

The first step in the causal chain involves *cooking behavior*, specifically stove adoption and use among households. The previous section detailed several possible barriers to stove adoption; use of new stoves is not guaranteed (even when they are distributed for free), and thus concerted efforts to measure stove use are a key component of an integrated measurement strategy. Use can be measured through surveys, which ask respondents about their cooking practices, as well as by instruments such as stove use monitors (SUMs) [26]. Each of these measurement approaches has its strengths and weaknesses. Surveys are subject to recall and social desirability bias (i.e., respondents may be reluctant to admit that they have not used new stoves provided by researchers), but allow collection of detailed, qualitative information on *why* stoves may or may not be used [27]. Meanwhile, SUMs allow cooking events to be estimated from a time series of stove temperature measurements, but require substantial effort in characterization of the temperature monitor/stove system. A combination of methods may thus be optimal to fully characterize stove use, yet few studies to date have paired comprehensive survey-based measurements with SUMs data collection. One notable exception is a study that examined a combined water filter and improved cookstove intervention in Rwanda using surveys and electronic sensors to measure use of both technologies [28]. In this case, households’ survey responses overreported the number of weekly cookstove uses measured by sensors by about 40%.

The next step in the chain involves the quantification of *cooking emissions* from the improved and traditional cooking methods. Many studies have measured real-time biofuel cooking emissions in laboratory settings using Water Boiling Tests or WBTs (e.g., [29,30]), but fewer have done field-based measurements [31-35]. Emission measurements in the field are essential since many key factors may vary between the lab and field setting (e.g., type and amount of fuels used).

*Household air quality* and *personal exposure* measurements shed light on the next steps in the causal chain. These measurements characterize the impact of changes in cooking technologies on pollutant concentrations in and around the home, and assess whether meaningful reductions in people’s exposure to these pollutants have occurred. To measure these impacts, studies have most commonly monitored concentrations and personal exposures to carbon monoxide (CO) and particulate matter less than 2.5 micrometers in diameter (PM_2.5_). Short-term CO exposure is associated with respiratory and cardio-vascular morbidity, as well as mortality, while long-term CO exposure has been associated with negative birth outcomes, developmental effects, and central nervous system effects, among others [7,36]. Personal CO exposure has been measured in the field with relatively cheap passive diffusion tubes for integrated concentrations, which require refrigeration, have moderately high uncertainty, exhibit batch-to-batch variability, lack the ability to capture peak exposures (in real-time) during cooking events, and have limits on length of deployment as maximum deployment measurement periods fall between one and two days [37-40]. Field studies monitoring PM_2.5_ exposure have faced logistical difficulties of obtaining subjects’ time-activity information and sampling continuously for longer than 24 hours. Cumulative PM_2.5_ filter sampling misses diurnal variations, and shorter sample durations lead to higher within-home variability [41] contributing to increased uncertainty in exposure estimates and intervention effects. Recent advances in monitoring and battery technology, as well cost reductions, have made it possible to measure PM_2.5_ at longer durations with smaller and quieter equipment. However, sometimes study participants are unable or unwilling to wear the air monitoring equipment as intended. Such breaches in protocol can lead to erroneous conclusions about personal exposure; therefore, it is essential to measure compliance.

This set of intermediate impacts is potentially linked to at least three key outcomes: *health burden*, *local to regional air quality*, and *climate change*. Given the large burden of disease that is linked to household air pollution, many cookstove studies conducted to date have focused on assessing health burdens using a large number of different health indicators. These include self-reported symptoms such as eye irritation and headaches [42], pulmonary function and respiratory symptoms [43], blood pressure and cardiovascular health [6], biomarkers of exposure to smoke-related compounds from urine [44], and biomarkers of systemic inflammation linked to smoke exposure from blood samples [45].

Emissions from cooking activities are not only important as household air pollutants and immediate personal exposures, but can also have detrimental impacts on air quality and climate at regional and global scales. Measurements of air pollutants, such as concentrations of PM and trace gases, can enable the quantification of the impact of cooking and biomass burning on regional air pollutant concentrations. For example, daily PM_2.5_ filter samples have been previously collected in Navrongo, Ghana from 2009–2010 [46]. Using source apportionment techniques, observed particulate elemental carbon (EC) and organic carbon (OC) and speciated elements were used to identify six sources of PM_2.5_, namely two-stroke engine combustion, diesel combustion, gasoline combustion, soil, biomass combustion, and road dust. After dust, biomass combustion was found to be second largest contributor to ambient PM concentrations in Navrongo [46]. Regional PM_2.5_ monitoring has also been performed in Accra [47,48], Nigeria [49], Ouagadougou, Burkina-Faso [50], Kenya [51], and Cairo [52].

To quantify larger-scale climate and air quality impacts, there have been several efforts to measure [33,53] and model the emissions from cooking activities around the globe (e.g., [35,54,55]). The contribution of emissions from cooking to the overall emissions burden of many important air pollutants, including particulate black carbon, is very large in many areas of the world. Further, these emissions can impact regional air quality and influence regional and global climate (e.g., [56]).

Individual steps in the described causal chain (Figure 1) have received varying amounts of attention in observational and intervention studies completed to date. Table 1 summarizes the types of measurements and analysis methods that have been included in some of the larger published stove intervention studies. One observation from this review is that some areas (e.g., exposure; health outcomes) have received considerably more attention than others (e.g., field-based emissions measurements, larger-scale climate and air quality). Two studies, the Patsari stove interventions in Michoacan, Mexico, and the Surya study in India (see Table 1 for citations), did incorporate measurements across all of the categories included in Table 1. However, there are important limitations in some of the measurements in both studies. For example, the Patsari study did not include any electronic stove use monitors, thus limiting the ability to quantify actual stove usage. Further, to date, the focus of the Surya study has been on black carbon emissions and this group has yet to include personal exposure to other pollutants or objectively measured health outcomes.

**Table 1:** **Summary of measurements included in prior randomized cookstove intervention studies**

**Name/Location of intervention study**	**Key publications**	**Intervention description**	**Types of Measurements Included**					
			**Stove use/acceptability**	**Emissions**	**Personal exposure**	**Micro-environment**	**Health**	**Regional air quality**
RESPIRE/CRECER Highland Guatemala	[24,37,40,57]	Collection of studies involved interventions with 500+ households using plancha improved stoves, gas stoves, and traditional (open fire) control groups	Quarterly stove use questionnaires; SUMs	Not measured in field	CO, PM_2.5_	CO, TSP, PM_10_, PM_3.5_, PM_2.5_	Blood pressure, acute illness (pneumonia), self-reported health symptoms	Not measured
Patsari/Michoacan, Mexico	[32,58-62]	Collection of studies involved interventions with 600 households using Pastari (ICS) and traditional (open fires) control group	Monthly visits reporting stove use	Field cooking tests (KPTs, WBTs and CCTs) and lab testing (WBT) in addition to GHG emissions measurements	CO, PM_2.5_	Kitchen/Indoor/Outdoor/Community Plaza for CO, PM_2.5_	Spirometry tests to measure lung function, blood samples, and self-reported health symptoms	PM_2.5_
Juntos and Barrick/ Peru	[44,63]	Two Intervention Programs; Juntos National (A), Barrick Gold Corp. (B) with 57+ households using improved custom brick stoves and traditional (open fire) group for baseline	Questionnaire & time use diaries at enrollment and 3 weeks after stove installation	Not measured	CO, PM_2.5_	Kitchen CO, PM_2.5_	Hydroxylate PAH biomarkers from urine samples	Not measured
DelAgua EcoZoom/Rwanda	[28,64]	566 households in three villages; EcoZoom Dura stove vs traditional. Intervention also included water filters	Surveys measuring acceptability and stove use conducted monthly for five months; SUMs on subset of stoves	No field measurements in Rwanda intervention study, but field-based emissions testing using same stove conducted in Uganda [34]	Not done in this study, but planned for follow-up	Kitchen PM_2.5_	Not done in this study, but planned for follow-up	Not measured
Surya/Indo-Gangetic Plains	[53,65-67]	Collection of studies involved interventions with 480+ households using a variety of improved biomass stoves, and traditional (mud/open fire) control groups	Surveys, Wireless Cookstove Sensing System (WiCS) (in development)	BC (Concentrations only)	Breathing zone BC	Kitchens/Outdoor BC and OC	Self-reported health symptoms	Regional BC and OC modeling
Ghana Sissala West	[38]	Intervention of 500+ households using constructed mud/brick stove and traditional (open fire) control groups	Surveyed participants on cooking activity and fuel wood gathering, SUMs	Not measured	CO	Not measured	Self-reported health symptoms	Not measured
India	[27]	Price experiment that tested 2 nontraditional cookstoves over 2,280 households.	Surveys used to access perceptions of stoves, health knowledge, socioeconomic status	Not Measured	Not Measured	Not Measured	Self-reported health symptoms	Not Measured
India	[13]	2,651 household intervention study subsidizing construction of inexpensive, locally-made mud stoves. Households responsible for providing mud, labor, and small payment for masonry and maintenance. Public lottery randomly assigned order of construction and distribution.	Three surveys in four years used to gauge stove usage, cooking activity, fuel expenditures, and perceptions about their efficacy	Not Measured	CO	Not Measured	Self-reported health symptoms, anthropometrics, spirometry tests to measure lung function	Not Measured
REACCTING, K-N District in Ghana	Work described here	200 household intervention study. Two types of biomass stoves introduced.	Surveys and SUMs	Controlled cooking tests in field	CO, PM_2.5_	CO and PM_2.5_ on a subset of homes	Biomarkers of inflammation from blood samples, anthropometrics, self-reported health questionnaires	Regional CO, NO, O_3_, and CO_2_ monitoring

The REACCTING (Research on Emissions, Air quality, Climate, and Cooking Technologies in Northern Ghana) study was specifically designed to include in-depth measurements along each step in the causal chain depicted in Figure 1. REACCTING is an ongoing interdisciplinary randomized controlled cookstove intervention study in the Kassena-Nankana District of Northern Ghana. The remainder of the paper details the study protocol and methodology. Results of this study are expected to generate novel insights regarding pathways towards improving public health and environmental quality in this region and beyond.

## Methods and study design

### Study area

**Figure 2:**
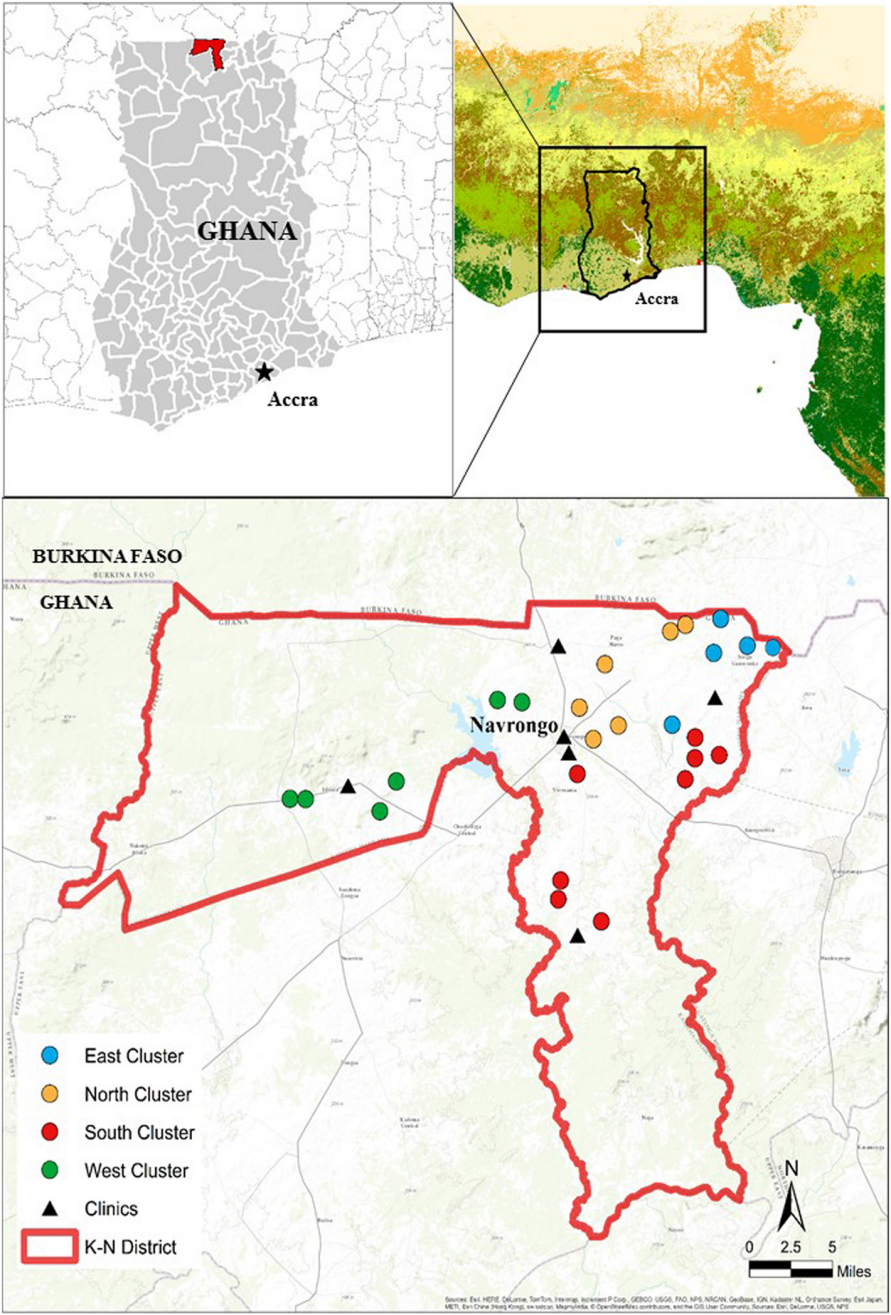
**Map of study area with cluster and health clinic locations.**

**Figure 3:**
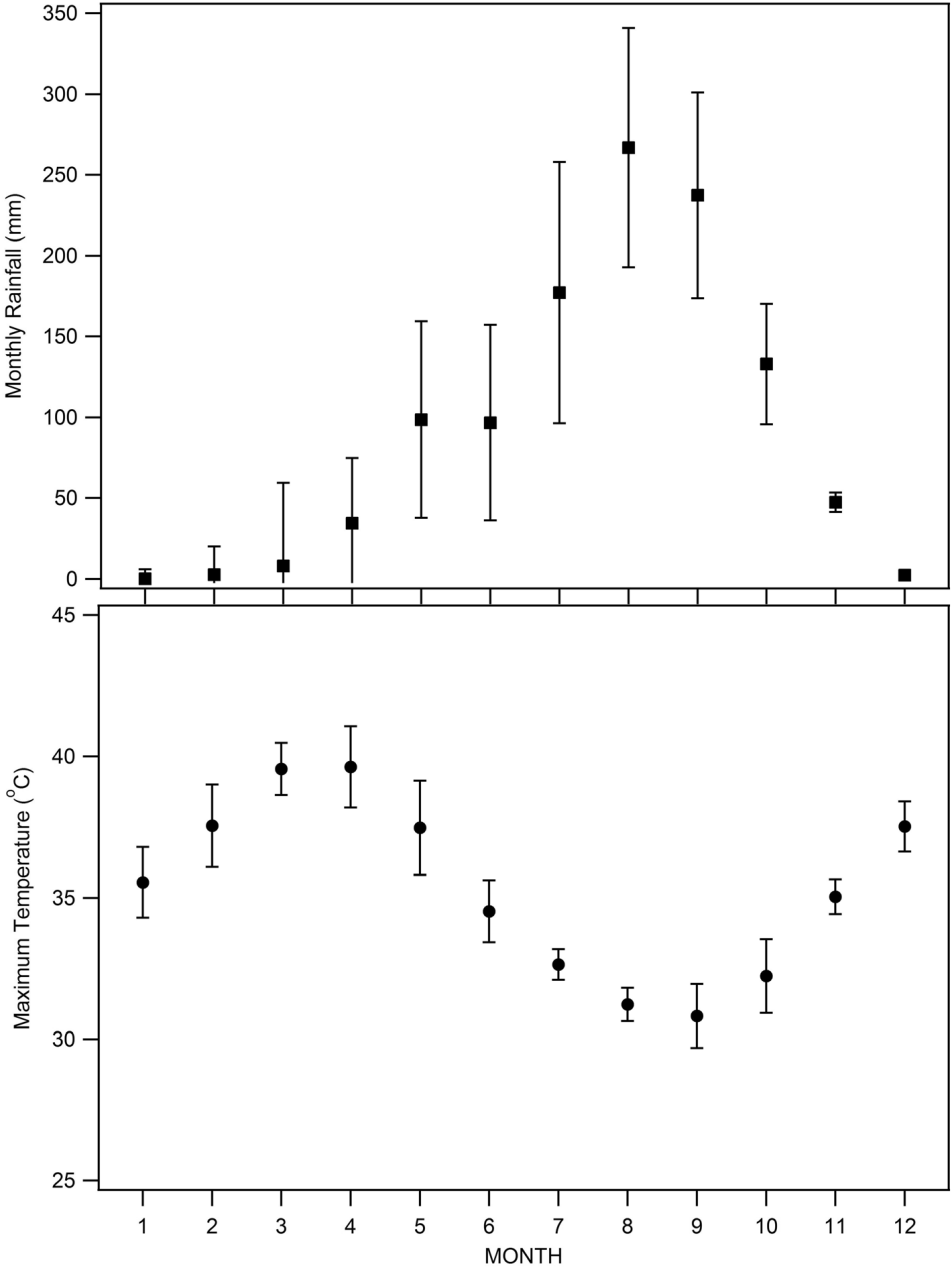
**Monthly rainfall and temperature in Navrongo.**

The REACCTING study is located in the Kassena-Nankana (K-N) District in Northern Ghana (Figure 2). This area has been described in detail by Oduro et al. [68]. Briefly, the district has a population of about 156,000 and an area of 1,657 km^2^. The climate in this region is generally hot and arid. A single rainy season lasts from approximately May to October, with more consistent rains occurring between June and September (Figure 3). The Harmattan, which typically occurs from late November through January, brings steady winds from the north with Saharan dust. This begins a dry season that continues until May. The K-N District is located in the northern savanna vegetation zone of Ghana dominated by woody shrubs and grassland. Much of the land is used for subsistence agriculture, with the dominant crop being millet.

The population of the K-N District is fairly homogeneous culturally. According to data from a district-wide Health and Demographic Surveillance Survey (HDSS) [68], about 80% of households in the district are located in rural areas, while 20% live in areas classified as urban. Among rural households, 88% report using biomass (wood or agricultural waste) as their main cooking fuel, while another 9% rely primarily on charcoal, and only about 3% of households cook primarily with gas or electricity. The traditional cooking method in this area is a three-stone open fire, and cooking is done in both indoor and outdoor areas. Ghana has one of the highest deforestation rates in Africa with the country’s forest an estimated quarter of its original size [69].

### Formative research

REACCTING builds upon a successful project done in 2010 involving a collaboration between the Navrongo Health Research Centre (NHRC), the National Center for Atmospheric Research (NCAR), and the University of Colorado-Boulder (CU). In that project, 222 households in Northern Ghana were surveyed to assess knowledge, attitudes, and practices as well as cost of illness associated with meningitis [70,71]. Motivated by studies suggesting a possible link between indoor cooking and meningitis [72,73], a follow-on pilot was conducted in 2011 to introduce efficient Envirofit G-3300 cookstoves to five families to explore acceptability and barriers to use in northern Ghana. Results from this pilot provided initial evidence of local acceptance of improved cooking technologies. Households that received the stoves were satisfied with their performance, finding that they were able to cook food faster and with less fuel than with their traditional open-fire stoves. Some problems were also reported with the stoves, mainly involving their stability when cooking a viscous porridge that is a local staple called Tuo Zaafi (TZ). The research team also observed that many households cooked with multiple stoves, including multiple three stone stoves (e.g., an indoor and an outdoor stove) as well as charcoal stoves. Building on this pilot, three additional types of woodstoves (EZY rocket stove, Philips gasifier stove, Gyapa rocket stove) were distributed to a total of 12 rural households in the K-N District between November of 2012 and March of 2013. Feedback provided by these households informed the subsequent design of the cookstove intervention and assessment that are described below.

### Sample selection

The REACCTING study includes 200 households for the stove intervention. This study sample was randomly selected from the population of the K-N District that met our study criteria using data (described below) from the district-wide Health and Demographic Surveillance Survey (HDSS) [68] and a cluster random sampling methodology. The social structure in this region is such that groups of related households live in connected compounds. Each compound is given a unique HDSS ID code, and this code is painted onto the wall of the compound and acts as a compound address. These codes consist of three letters (the cluster ID), the first of which denotes the cluster’s geographical region within the district (North, South, East, West, and Central), and a two-digit compound number. Household IDs are then assigned within each compound.

The target population for this intervention was rural households in the K-N District that use biofuels (wood, animal waste, and crop residue) as their main cooking fuel sources. Within these rural households, we focused on those individuals in closest proximity to cooking activities: women and young children. Thus, we used a set of cluster- and household-level criteria to generate a subpopulation of eligible households from which to randomly draw our study sample. To generate this subpopulation, we first eliminated all clusters in the primarily urban “Central” cluster, as well as other clusters in which more than 25% of households were classified as urban in the HDSS. For logistical reasons, we also eliminated a small set of clusters that were deemed to be difficult for interviewers to access. Since the intervention was rolled out at the cluster level, as described in more detail below, we also dropped all clusters that had less than 10 eligible households after all of the household-level eligibility criteria were applied. At the household level, to ensure a relatively uniform, rural sample of households, households that did not list biofuels as their main cooking fuel and households that did not use boreholes as their main water source were eliminated. Finally, we included only households with at least one child under five and one woman between the ages of 18 and 55.

Using this subpopulation, sample selection proceeded in two phases. First, we randomly selected 25 clusters using population weighting to determine the number of clusters selected per region: five clusters were randomly selected from the East, six from the North, eight from the South, and six from the West (Figure 2). Next, ten households (eight primary households and two alternates to be used if the primary households could not be enrolled) were randomly selected from the population of eligible households in each of these clusters. Since cooking duties may be shared within compounds and emissions from one household’s cooking could affect exposure and health outcomes of other households within the compound, we included a maximum of one household per compound. In cases where there were multiple eligible households in a compound, we randomly selected only one for inclusion in the sample. Given this sampling methodology, our study sample can be said to be representative of the subpopulation of the K-N District that meets our eligibility criteria: rural, uses biofuels as their main cooking source, and has women and young children in the household. Overall, this subpopulation from which our sample was selected includes 59% of all clusters in the district (194 out of 331) and about 20% of all households in the district (5,918 out of 29,403).

### Ethical review

The study protocol was reviewed and approved by the Human Subjects Committee at the National Center for Atmospheric Research and the Institutional Review Board of the Navrongo Health Research Centre. Informed consent was obtained from all study participants prior to any data collection. Oral consent was obtained for the household survey, personal exposure monitoring, and household environmental monitoring, and written consent was obtained for the subclinical health measures (anthropometrics and blood spots). (See “Assessment of intervention impacts” subsection for full description of these measurement techniques). For the measurements conducted with children (personal exposure and subclinical health measures), consent was obtained from each child’s parent or guardian.

### Community entry

A series of community entry activities were undertaken by the research team in order to inform community members about the research project and to obtain local leaders’ permission to carry out the proposed research activities. NHRC investigators with extensive knowledge of the local context and norms led this effort, which involved meetings in all of the 25 clusters selected for inclusion in the study. These meetings involved local chiefs, community elders, opinion leaders, and women’s groups. These meetings also served to address any concerns participants may have had and to foster trust in the study’s objectives and fairness.

### Stove technologies

**Figure 4:**
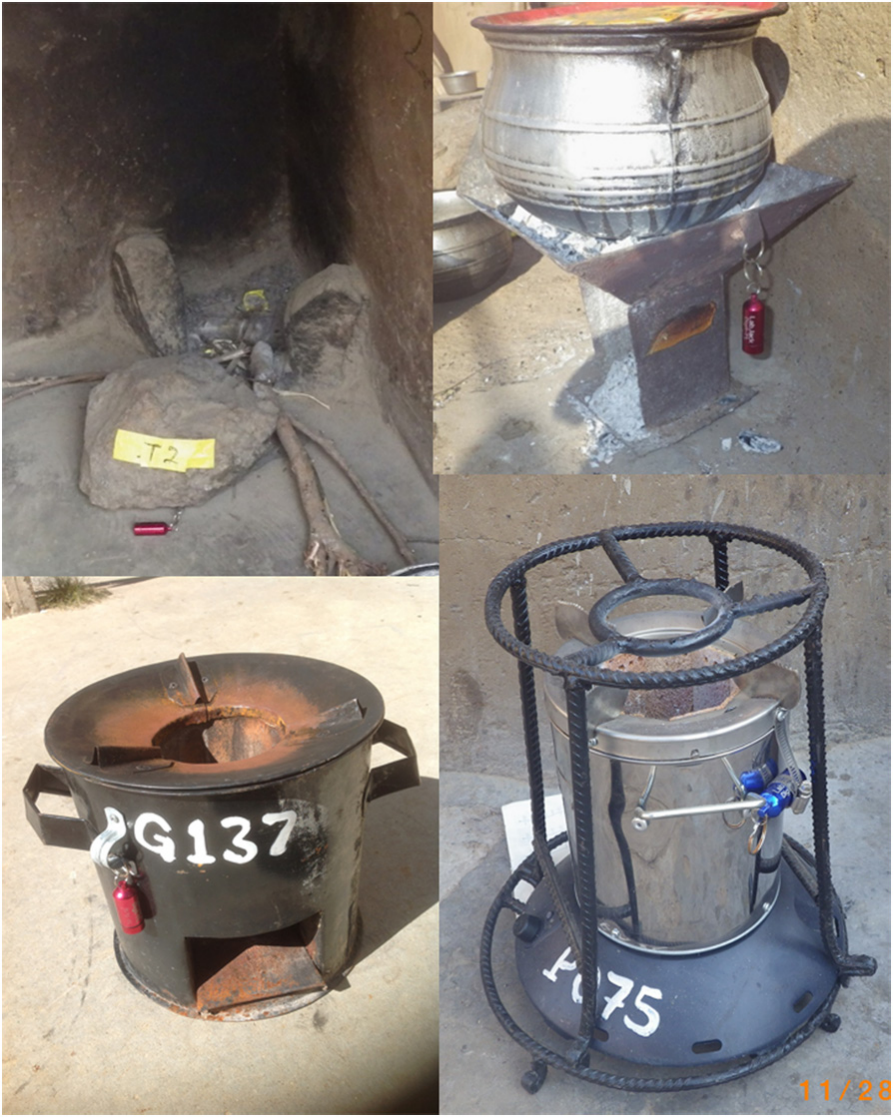
**Traditional and improved stove technologies being compared in the REACCTING study, shown with Stove Use Monitors (SUMs) attached.** Top left: traditional three-stone stove. Top right: traditional charcoal stove. Bottom left: Philips Smokeless Stove, Made in Lesotho (Southern Africa), Cost: ~US$125. Bottom right: Gyapa Wood-Burning Stove. Made in Accra. Cost: ~US$15-25.

The selection of cookstove technologies for this study was guided by a number of considerations. Based on extensive feedback from households in the K-N district who tested several stove models during the pilot phase (2012–2013), the Philips Smokeless Woodstove and the Gyapa Wood Stove (Figure 4) were deemed to be potentially promising technologies for this population. The former is a gasifier stove produced in Lesotho. This stove is visually perceived as “high-tech”, requires power to perform properly, and has been observed to be a low emitting technology, Tier 4 stove, during lab testing [29]. The latter was designed and locally manufactured specifically to fit the cooking needs of the study population; this process is described below. These two stoves also represented two distinct rungs in the stove “ladder”. On the lower rung, the Gyapa stove is locally produced, affordable, and more fuel efficient than three stone fires, though not expected to drastically reduce cooking emissions. The Philips stove represents a higher-rung stove: it is widely believed to be among the cleanest biomass-burning stoves available and has been used in other intervention studies (e.g., http://www.projectsurya.org/). The Philips stove is also substantially more expensive than the Gyapa stove and must be imported into Ghana. Comparing these two stoves side by side in the same population thus presents an opportunity to generate novel data to inform the international debate between those advocating incremental versus transformative approaches to tackling the cookstove challenge.

The Gyapa Wood Stove was specifically designed for use by populations in the Northern Regions of Ghana by Relief International/Gyapa Enterprises (RI/Gyapa). RI is a Los Angeles-based global humanitarian organization that employs a team of 2,000 relief and development professionals working to bridge the gap between immediate emergency relief and long-term community development. As part of its Social Enterprise program in Ghana, RI supported production of the Gyapa Fuel –Efficient Cookstove, a locally produced and distributed improved cookstove sold primarily for charcoal burning households across Ghana. The Gyapa charcoal stove is the most popular improved cookstove in Ghana and is comprised of the largest improved cookstove production industry on the continent. Since Gyapa’s inception in 2002, over 600,000 stoves have been locally produced and sold in Ghana.

RI/Gyapa joined the REACCTING study team after the project was funded, at the stage of the project when stove technologies were being evaluated for inclusion in the study design. They subsequently designed and produced the Gyapa Wood Stove to fit the needs of rural populations in the north of Ghana, and also provided input and training on the stove distribution and education components of the project. To develop the Gyapa Wood Stove, RI/Gyapa developed and tested several prototypes in Accra as well as in Navrongo to determine user preference, applicability, required durability and suitability for the study. A similar model was used in a past intervention study in Accra, and saw significant decreases in kitchen CO and PM_2.5_ levels [74]. Multiple iterations of test stove designs were produced and tested with wood burning communities in Accra. Tests included respondent likes/dislikes about the models, perceived fuel usage and smoke emissions as well as eye and throat irritation, cooking time, and comfort while cooking. Stove tests also used similar sized pots for cooking as are used in regions of Northern Ghana. Respondents perceived the air quality in the cooking areas as better and reported less smoke emission and less exposure to heat associated with the use of the improved wood stove prototypes as compared to traditional wood stoves. Stove manufacturers used this feedback along with knowledge of combustion efficiency and local supplies and skills to generate a final prototype.

The final prototype of the Gyapa Wood Stove included a combustion chamber, often called a rocket-stove design, with a ceramic liner on the inside and an outer liner of insulation and saw dust to increase heat retention. The additional insulation also creates a heat barrier that reduces heat on the external parts of the stove to prevent burns when handling the stove. The Gyapa Wood Stove was produced by contracted ceramist and metal artisans who are a part of the Gyapa network. The producers of the woodstove model, as beneficiaries of improved stove models themselves, also brought intimate knowledge of Ghanaian cooking habits and cultures, which supported the design process.

In addition to designing and manufacturing the Gyapa Wood Stove, RI/Gyapa worked with the study team to design and produce a pot support structure for the Philips stoves (Figure 4). These stands were made of rebar and fit around the Philips without modifying its design or function. The stands provide more stability and enable the accommodation of larger pots in order to make it more culturally appropriate for local cooking practices.

### Stove intervention design

The stove intervention of the REACCTING study includes four different intervention arms: *Group A* received two Gyapa stoves, *Group B* received two Philips stoves, *Group C* received one of each type of stove, and *Group D* serves as the control for the duration of the study, but will receive their choice of stove at the conclusion of the study. Stove stacking (i.e., households using new cookstoves alongside traditional cooking methods) had been observed in prior studies and we had earlier observed use of multiple stoves among households in the study area. Thus, two stoves were provided to each intervention household to increase the probability that households would begin to substitute away from traditional stoves rather than simply adding a new stove to their cooking technology mix. Because households may prefer using different types of stoves for different purposes (e.g., cooking TZ with the Gyapa stove but rice or soup with the Philips), one study group (Group C) has been provided one of each stove.

Small meetings involving study participants from one or two clusters (8–16 study participants) were used to educate participants about the two new stove technologies, inform households about the study design and objectives, randomize households into different treatment groups, and distribute the stoves. These meetings were held in November-December, 2013, between one and five days after the study households were initially contacted for the study’s baseline survey (described in detail in the next section). Meetings were held at a central point within the cluster such as a school or a market. A representative from each household in the cluster attended the meeting; usually, this was the survey respondent (primary cook). However, if the survey respondent was not available, another household member attended in her place.

Education and outreach are essential to ensuring take-up and appropriate use of any new technology. As such, several steps were taken to ensure that participants were given accurate information about the different stoves, including how and why to use them. Retired female nurses from the K-N District, who spoke the local languages and were known and trusted by community members, were enlisted as stove ambassadors. These ambassadors and other members of the stove distribution teams were trained in stove use, including the best way to feed the stoves with fuel wood in order to increase the stoves’ thermal performance and reduce smoke, as well as effective outreach by our partners from RI/Gyapa, who have extensive experience with stove promotion. In other cookstove work, RI has found that marketing stoves as an “aspirational” product has been more successful than focusing on health impacts alone. That is, uptake of stoves may be greater when they are promoted as status symbols, or when other benefits such as convenience, faster cooking times, and fuel/cost savings are emphasized. While this message was delivered as part of the training RI provided to the intervention team, the particular make-up of the study team in this case (via the Navrongo Health Research Centre) resulted in a more health-focused message. We acknowledge this as a potential limitation of our approach.

During the stove distribution meetings, the ambassadors and stove distribution team members led a demonstration of both types of stoves and gave participants the opportunity to inspect the stoves and ask questions. During the meeting, team members also explained the study design to participants, including the fact that different households would receive different types of stoves so that the research team could assess which stove or stoves worked best, and that some households would not receive new stoves until the conclusion of the study so that researchers could compare what happened in households using new stoves with those using traditional stoves. Participants were told that these households would have their choice of stoves at the end of the study.

Following the stove demonstration and explanation of the study, each participant drew a slip of paper with a letter (A, B, C, or D) representing their intervention group. For each cluster of eight households, two households were assigned to each of the four intervention arms. Participants in Groups A, B, and C received their stoves at the meetings, and all participants were given matches and a pair of iron bars for bracing pots while cooking. While we considered conducting the randomization electronically prior to the meetings, we ultimately decided that having the participants draw their groups themselves during the meeting increased the transparency of the randomization process. Indeed, following the meeting, interviewers reported that households that selected into the “D” group expressed disappointment, but that they saw the process as fair and legitimate and remained committed to being a part of the study.

Within a week following the initial stove distribution, stove ambassadors and other team members visited households to provide additional training opportunities on appropriate usage as well as to answer questions; the objective was to ensure that participants felt as comfortable as possible with the use of their new stoves.

### Assessment of intervention impacts

Several assessment methods are being used to measure indicators at multiple points along the causal chain shown in Figure 1.

#### Cooking behavior

Cooking behavior and cooking technologies are closely linked; we cannot understand the real world impact of a cooking technology if we do not understand how that technology alters behavior. In all 200 households, a series of detailed household surveys are being conducted to assess cooking behaviors, among other important social, economic, and behavioral outcomes. Surveys are administered in the local languages of the district (Kasem or Nankam) by native speakers of each language. A baseline survey was conducted in all households prior to stove distribution (Nov-Dec ’13). This survey took approximately one hour to complete, and measured household composition and demographics, attitudes and priorities, cooking behaviors, knowledge and perceptions of health and environmental issues related to cooking practices, demand for new stoves, and self-reported health symptoms. To assess cooking behavior, the respondents gave detailed information about the number and type(s) of stoves used, type(s) of fuel used, types of food cooked, as well as who cooked within their household. The baseline survey also included a detailed economic choice experiment exercise aimed at measuring demand for new cooking technologies and willingness to pay for specific stove attributes such as smoke reduction, reduced fuel use, and shorter cooking times. Follow-up surveys were completed in March, May/June, and August of 2014. The follow-up surveys are substantially shorter than the baseline (approximately 20 minutes) and focus primarily on cooking behaviors as well as self-reported heath symptoms. Additional surveys are scheduled for December of 2014 and April and November/December of 2015. These longitudinal surveys will track use of the different stoves (both old and new stoves) over time, including differences across seasons. For example, we expect to observe more indoor cooking during the rainy season. Stove preference and willingness to pay responses will also be compared over time to assess how these measures change as a function of a household’s experience with the new technologies.

Survey-based measurements allow respondents to provide detailed information about a range of factors, including rich qualitative information about why stoves have been used or not. However, self-reported stove use data are also subject to measurement error due to recall bias as well as social acceptability bias (i.e., respondents may not want to offend researchers by telling them they have not used their new stoves). Thus, in addition to reported stove use information gathered through the household surveys for all 200 households, stove usage is being monitored electronically in a randomly selected subset of 35 study households from the four different intervention groups. In this subset, Stove Use Monitors (SUMs, Labjack Digit-TL) are attached to stoves and continuously measure temperature, such that stove use can be assessed by observing an increase in temperature in excess of ambient. Placement of the SUMs was tested in the lab prior to the study with the two intervention stoves. In the intervention groups (Groups A, B, C), both new stoves and the most-used traditional stove are monitored. In the households of the control arm (Group D), the two most-used traditional stoves are monitored. One-minute data for each SUMs deployed in the field is being collected every 3–4 months.

SUMs have been used previously to assess stove adoption [26,75]. As others have noted [26], monitoring cookstoves can be challenging due to variability in usage behaviors and varying stove thermal mass, leading to different temperature profiles. This is especially true of the three-stone fires, as they often have slower heating and cooling times, and the stone arrangements can vary substantially [76]. Cooking event detection and cooking time estimates will be calculated using methods described by Ruiz-Mercado et al. [26] and Simons et al. [76], as well as methods developed using our own observational data.

#### Cooking emissions

**Figure 5:**
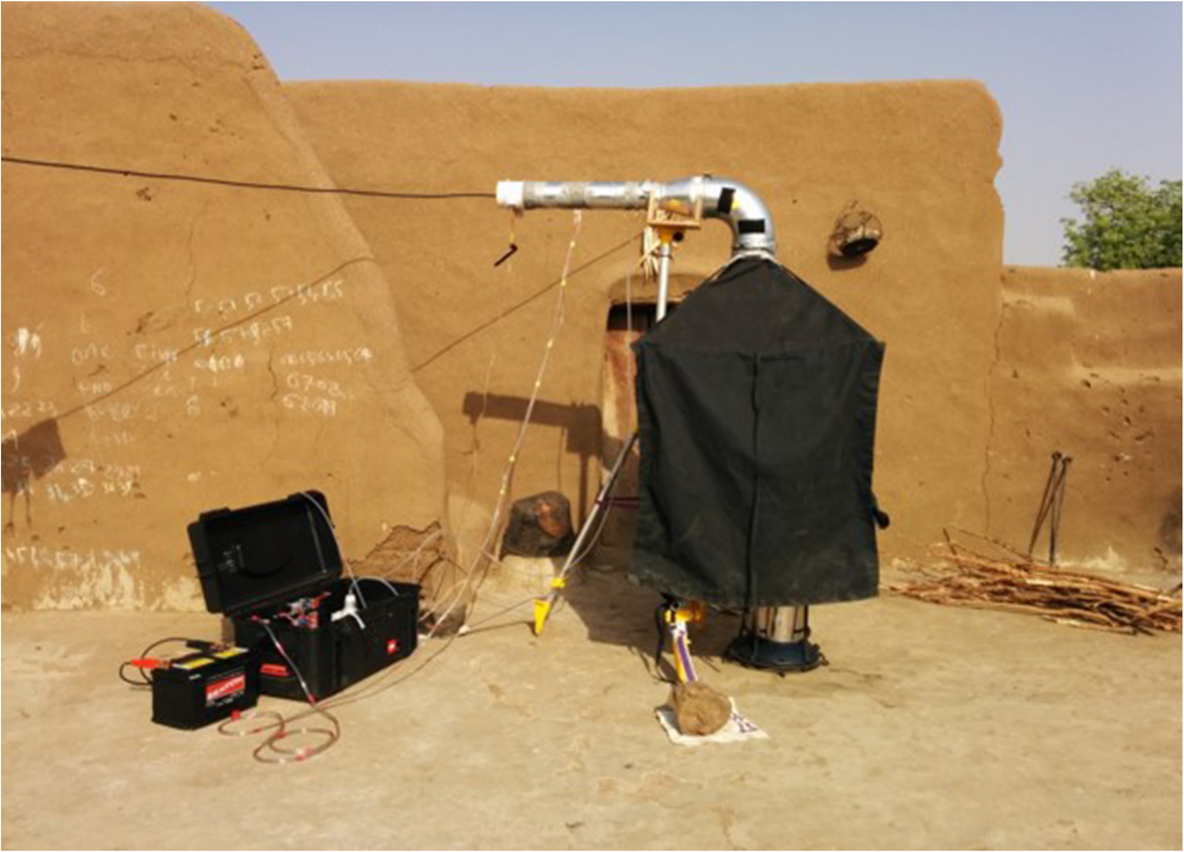
**E-Pod setup for measuring in-field stove emissions.**

Real-time cooking emissions are measured in-field using a modified controlled cooking test (CCT) [77]. We are measuring 15–20 samples from each of the three main stove types (Gyapa, Philips, three-stone). To measure the emissions, we developed a monitor (E-Pod, Figure 5) similar to the portable emission measurement system (PEMS) designed by Aprovecho Research Center [78]. The E-Pod uses low-cost sensors to measure real-time carbon monoxide (CO), carbon dioxide (CO_2_), nitrogen oxide (NO), nitrogen dioxide (NO_2_), total volatile organic compounds (TVOCs), temperature and relative humidity. CO, NO, and NO_2_ are measured with electrochemical sensors (Alphasense B4), with CO also measured with a metal oxide (MOx) semiconductor sensor. CO_2_ is measured using a non-dispersive infrared (NDIR) sensor, and TVOCs are measured with a photoionization detector (PID Silver, Baseline-Mocon). Total particulate matter (PM) is collected on a quartz fiber filter for subsequent analysis of elemental and organic carbon (EC/OC) as well as organic molecular markers. These analyses allow us to further understand the chemical nature of the exposures as well as derive the origin of the particles. For each household selected, emissions are measured during the entire cooking process, starting from 15 minutes prior to lighting the fire and ending 15 minutes after the fire is out. A typical emissions observational period takes 2 to 4 hours, depending on the type of meal that is being cooked. One of the primary meals is the thick millet flour porridge called TZ, which is prepared by boiling water, adding flour, then simmering and stirring vigorously until there is a dense smooth porridge. This process typically takes 30 to 45 minutes. This starchy staple is usually eaten with a vegetable soup. Woody biomass is the most common fuel used in the study area, with charcoal (charred woody biomass) occasionally entering the mix. The biomass is from different trees found in the area such as neem (*Azadirachta indica*), sheanut (*Vitellaria paradoxa*), and mango (*Mangifera indica*).

#### Personal exposure and household air quality

Personal exposure and household air quality are measured in the participating households throughout the study period. These specific measurements are made approximately once per year in the participating households, and for the 35 households outfitted with SUMs, these measurements are made approximately four times per year. The additional measurements coordinated with the SUMs enable the characterization of the SUMs relationship with exposure, as well as the within-household variability.

To assess personal exposure to pollution from biomass combustion during a household’s monitoring period, real-time CO monitors (EL-USB-CO300, Lascar Electronics) with a one-minute resolution are worn by the survey respondent (primary cook), children under five, and as many other household members as are willing. CO has previously been explored as a surrogate for PM_2.5_ exposure from biomass combustion [40,57,79]. CO is relatively straightforward to quantify continuously and requires fewer resources compared to PM_2.5_. Past work often used adsorption tubes for integrated CO exposure assessment, but recent advances in sensor technology have made it possible to use real-time wearable electrochemical CO monitors [40,80-82]. Such monitors are simple to operate and have a long battery life. Electrochemical CO sensors in general demonstrate low inter-sensor variability, and moderately good zero and span stability. However, the sensor dynamics, specifically response times to changes in concentrations, have not been well quantified.

Adults wear the CO monitors around their necks using a lanyard, and children below age eight are given specially designed t-shirts with pockets sewn on the lapel. Forty-eight hour monitoring periods are employed to account for day-to-day variability. The monitoring periods typically begin on Monday, with distribution of CO monitors to four households of a given cluster, one from each study arm. The monitors are then collected on Wednesday and redistributed to a set of four households from a different cluster, until Friday when they are picked up for calibration over the weekend.

During each sampling period, one of the four households is selected for supplementary measurements: personal PM_2.5_ (only for participants over the age of four), step counting with pedometers, and microenvironmental monitoring in the cooking area. The personal PM_2.5_ monitors are worn in small backpacks or fanny packs. These monitors collect particles on quartz filters using an impactor (25 F-2-2.5, URG Inc.) and pump (Airlite, SKC Inc.) for EC/OC and organic molecular marker analysis. Resulting PM_2.5_ samples are integrated over the 48-hour monitoring period. In these focus households, a microenvironment monitor called a G-Pod (mobilesensingtechnology.com) is also used to measure CO, CO_2_, and PM_2.5_ in the cooking area during the monitoring period. The G-Pod CO measurement uses the same electrochemical sensing principal as the Lascar CO monitors. CO_2_ is measured with a low-cost NDIR sensor (S200, ELT Corp.), and cumulative PM_2.5_ is collected using a quartz fiber filter with a 2 liter per minute flow rate. The G-Pod is placed one meter off the ground, and one meter away from the most-used cookstove. In a subset of these households, near-continuous PM and temperature sensors (University of California at Berkeley Particle and Temperature Sensors or UCB-PATS, [83]) are deployed as well, to supplement the integrated PM_2.5_ data. Each household sampling visit concludes with a short stove usage survey, intended to identify those in the household who have cooked, the stoves that were used, the meals that were cooked, and the fuel used. Participants are also asked to provide an estimate of the fuel that will be used the following day. If an estimate is provided, the fuel is weighed and moisture content is measured.

The relationship between microenvironmental air quality and personal exposure is highly dependent on individuals’ time-activity patterns, namely when and how often participants are in close proximity to emission sources. Thus, in a subset of at least 10 households from each study arm, proximity measurements are being collected using a method similar to Allen-Piccolo et al. [84]. Bluetooth LE beacons are placed in the G-Pods (placed near stoves), and individuals carry a mobile phone with a custom Android application during personal exposure measurements. The mobile phone receives the Bluetooth signal emitted by the beacon, and the strength of that signal is then roughly translated to a distance measure from the person to the source. In addition to measuring proximity to the G-Pods at the homes, the mobile phones in the packs use GPS to record global position. This information can further be applied to estimate distances to other emission sources that are identified in the region.

#### Health burden

Three methods are used to measure human health outcomes as part of the REACCTING study. The household survey includes questions about *self-reported health symptoms,* including respiratory symptoms, for the respondent (primary cook) and all children under five in the household. Subclinical health measures are also collected twice a year to provide data on more continuous indicators of these individuals’ health status. The two types of measures include *anthropometrics* and *biomarkers of inflammation*. Anthropometric measurements of height, weight, and mid-upper arm circumference serve as indicators of an individual’s nutritional status, which in turn can be affected by acute and chronic illnesses [85]. While we are not aware of any studies directly linking cookstove exposure to these child growth measures, we hypothesize that lowered exposure to cooking emissions over time may result in better growth outcomes. Hanna et al. [13] made similar measurements in their cookstove intervention study in India. (Results were not significant in this study, which is not surprising since use of the improved stoves was low). Meanwhile, biomarkers are measured from blood spots taken from study participants at the times of the major surveys (Nov-Dec ’13, May-June ’14, Nov-Dec ’14, and May-June ’15). The markers targeted in the analysis include: C-reactive protein (CRP), Serum Amyloid A, soluble cell adhesion molecules (sCAMs), including sICAM and sVCAM, interleukins (IL-1B, IL-6, IL-8), and tumor necrosis factor alpha (TNF-a). These markers are chosen as they indicate the presence of systemic inflammation or vascular injury, and perturbation in their regulation may be a risk factor for cardiovascular disease. Previous epidemiology and clinical studies have shown associations of nearly all of these markers with exposure to particulate matter [45,86,87]. The biomarker data will thus enable assessment of potential changes in systemic inflammation over time for each individual as well as across individuals as a function of changing levels of smoke exposure.

#### Regional air quality

To understand the spatial and temporal variability of air pollution and to help identify pollutant sources, G-Pods are deployed throughout the study region. Such regional monitoring has been undertaken in developed countries, but very rarely in developing countries (for example, see Mead et al. [88]). The G-Pods are configured to measure O_3_, CO, NO, and NO_2_ using Alphasense B4 electrochemical sensors. Ozone, CO, and NO_2_ are also measured using MOx sensors from SGX Technologies. CO_2_ is measured using the previously mentioned NDIR sensor. Some of the G-Pods also measure TVOCs using photoionization detectors, as well as wind speed and direction. The G-Pods are mounted three to four meters above ground at the five Ghana Health Service clinics in the K-N district: Paga, Kandiga, Kologo, Chiana, and the Navrongo Health Centre (NHRC) (Figure 2). The NHRC also serves as the study core monitoring site and, in addition to the low-cost monitors, reference quality instruments are operated there. At the NHRC, CO is measured with a Thermo Model 48 CO analyzer set to re-zero every hour using a heated Pt-Al catalyst. Ozone is measured with a 2B Technologies Model 202, while NO and NO_x_ are measured with 2B Technologies Models 401 and 410. CO_2_ is measured with a LI-COR 840a. Weekly PM_2.5_ filter samples are also collected on 90 mm quartz fiber filters using a cyclone (30E, URG Inc.) and filter holder, employing a flow rate of 5.5 liters per minute maintained with low-power vacuum pumps audited monthly with a rotameter and checked daily with a flow totalizer and timer. The PM_2.5_ sampling will expand upon the work of Ofosu et al. [46], and filters are analyzed for EC/OC and organic compounds. Meteorological data are collected using a Climatronics sonic anemometer and temperature and humidity sensor. Additionally, pollution source sampling is conducted for a variety of common emission sources in the region including trash burning, different types of commercial cooking, and vehicle emissions. The locations of major sources will also be identified in order to inform analysis of individuals’ proximity to these sources.

### Analysis and integration

The REACCTING study was designed to provide robust and integrated measurements at each stage in the causal chain linking an improved cookstove intervention to key outcomes of interest (Figure 1). Our methods are informed by prior studies and assessment strategies, drawing lessons from the strengths and weaknesses of those experiences. To assess each intermediate and final outcome, we typically employ multiple measurement strategies rather than relying on a single source of data. This redundancy in data sources enables our research team to analyze and integrate across data streams to provide more detailed and nuanced answers to the key research questions. Three examples of these integrated analyses are presented below.

#### Integrated stove use analysis to understand cooking behaviors

Understanding how, why, and which types of stoves are used by the study households is a crucial first step in analyzing and interpreting subsequent outcomes (e.g., emissions, air quality, health) in the causal chain. Data from household surveys and electronic SUMs are analyzed jointly to provide comprehensive information about stove usage, activity, and preferences. Survey data are available at approximately three month intervals throughout the first year of the study, and six month intervals during the second year. The survey data contain information about every stove in all 200 study households, including traditional and improved stoves. Reported use of each stove in the week prior to the survey (number of days on which each stove was used), on the day prior to the survey, and at the time of the survey are collected. Types of fuel used and dishes cooked on each stove are also recorded. Meanwhile, SUMs measure stove temperature every five minutes for a subset of households in each intervention arm. Within these households, measurements are available for improved stoves as well as the most-used traditional stove. The integration of the survey and the SUMs data streams provides robust information about any reductions in three-stone stove usage among the different intervention arms. Comparing results between the surveys and the SUMs will also determine whether households tend to over-report use of new stoves in our study, as was found in a similar comparison in the context of a stove intervention in Rwanda [28]. Finally, quantifying behaviors, such as the dishes cooked and the perceptions of stove quality and performance, in addition to the amount of stove use will help to point the way forward towards interventions and scale-up efforts that can be piloted in future studies to further increase stove acceptability and use.

#### Integrated measurements to assess the contribution of multiple emissions sources to personal exposure

The REACCTING study, like other cookstove interventions, directly targets a key source of pollutants to which individuals are exposed: cooking emissions within the home. However, other sources of emissions, such as vehicle emissions and trash burning, also contribute to local and regional air quality, and thus personal exposures to air pollutants. Integrating data across emissions, personal exposure, microenvironmental, and regional air quality measurements will allow us to better understand the personal exposure contribution of household cooking, along with these other emissions sources. One way in which this will be accomplished is with the use of source apportionment of the personal and regional PM_2.5_ organic molecular markers. This technique uses the covariance of chemical tracer species to apportion a set of measurements into matrices of chemical compositions and contributions, termed factor profiles and contributions, respectively. Thus, we will identify organic PM_2.5_ factor profiles in both the personal and ambient samples and learn the impact of each on exposures and ambient air quality. The source emissions measurements will help validate the personal exposure source apportionment results. In addition, comparison of the profiles generated using the ambient and personal filter samples will shed light on the validity of using ambient measurements to understand personal exposure in the region.

Since the number of personal PM_2.5_ exposure samples are constrained due to resource limitations, we will also develop models to predict PM_2.5_ exposures based on the easier to collect microenvironmental and regional samples. Household microenvironment air quality monitoring has been performed as a proxy for personal exposure with mixed success [6,39,41,57,89]. This approach can help predict personal exposure and assess local impacts, but can require time-activity logging to reliably estimate exposure with increasing numbers of pollution sources (e.g., [39-41]). To predict time-integrated PM_2.5_ information from different data streams, we will use real-time time-activity data to apportion users’ exposure time to the microenvironment and ambient PM_2.5_ samples. In the subset of samples with UCB-PATS monitors, the microenvironment PM_2.5_ sample will be weighted by the real-time PM measurement from the collocated PATS to provide a better estimate of the contribution by taking the microenvironment PM dynamics into account. We will also predict personal CO exposure following a similar procedure, but using the higher time resolution CO at the personal, microenvironment, and regional scales. Models have been developed to relate personal and microenvironmental measurements in past cookstove studies [6,41], but not with high-resolution time-activity data, nor in this region. As done in other works [39,40,90], we will also investigate the PM_2.5_ vs. CO relationship at the personal and microenvironment levels.

#### Integration of field measurements to develop regional emissions scenarios for air quality modeling

To assess the impact of cooking on air quality and climate, emissions from this particular source must be quantified. Emissions of PM (including organic and black carbon), CO, and other reactive and greenhouse gases from cooking are a function of activity and emission factors (or the mass of emitted pollutant per time cooked or fuel used). Emission factors are based on the stove and fuel used. Activity is the information that describes cooking practices, such as the timing and duration of cooking and the types of dishes prepared.

The information collected as part of the REACCTING surveys and the emissions, SUMS, and microenvironment measurements will provide the basis for the development of an emissions inventory for current and potential cooking activities in the region. Emission factors are developed from the emissions measurement experiments during which emissions are directly measured and normalized to the amount of fuel burned. These in-field measurements will be compared to published laboratory measurements with the goal of improving our ability to predict emissions from alternative cooking technologies and fuels. As previously discussed, the activity information collected via the SUMs will provide data to constrain the typical timing and duration of cooking events, and can be combined with the survey information to assess typical cooking practices, such as the frequency of specific dishes and meals. These data enable the determination of when the emissions occur, for how long, and under what conditions. Together, the emission factors and activity information will be used to produce hourly estimates of emissions from traditional cooking practices, as well as from cooking activities that use the technologies introduced in the REACCTING study. The estimated household emissions inventories will then be scaled to the greater regional population using the HDSS data.

The scaled-up emissions estimates will be used as inputs into chemical transport models, such as the Weather Research Forecasting model with Chemistry (WRF-chem) [91], which simulate the coupled interactions between regional weather and emissions from cooking activities and other sources. WRF-chem will thus serve as a tool to scale up our field results by simulating the regional air quality (O_3_, CO, PM) across a broader region of western Africa that will encompass the study area in northern Ghana. Emissions sources in the simulations will be developed from the field-based cooking and regional air quality emissions measurements described above, as well as from existing emissions inventories (e.g., [92]). Biomass burning emissions will be estimated from the Fire Inventory from NCAR (FINN) model [93]. WRF-chem simulations will be performed for a variety of cookstove adoption scenarios (e.g., widespread adoption of Gyapa stoves, of Philips stoves, of both types of stoves, no adoption of clean cookstove technologies, etc.) based on the results from the surveys and for both historical and future climate change scenarios. The results will be used to more thoroughly examine the ambient exposures to which the regional population is exposed, and how that might change with different technologies and behaviors. Further, the models can be used to assess the comparative impact of local behavior and technological changes versus regional climate variability and change on local air quality and health outcomes.

## Discussion

The practice of cooking with biomass over open fires is widespread throughout much of the world. In many ways, this is understandable: this cooking method is low-tech and requires few resources beyond locally available materials (biomass, stones) and the time of household members (for collecting fuel and preparing food). While there are many reasons to believe that shifting cooking practices could have wide-reaching benefits for some of the world’s most disadvantaged populations, achieving this objective in practice requires well-designed interventions that understand and integrate existing cultural practices.

The REACCTING study represents an attempt to systematically address some of the challenges that previous cookstove studies and interventions have faced in order to generate multidisciplinary and detailed data that can be used to inform broader efforts to change cooking behaviors in this region and elsewhere. The cookstove intervention we have implemented employs both high- and low-tech biomass stove options in order to inform the debate between those advocating transformative approaches and those arguing that incremental progress is more feasible and will achieve more in the long run. In addition, we distribute two stoves to each household assigned to an intervention group in our study. This is intended to address the potential for stove stacking. Formative research in the area showed that prior to any intervention, households were using multiple stoves and a mix of technologies to meet their cooking needs. By providing households with multiple new stoves, and in some cases two types of new stoves (Group C), we hypothesize that households will begin to substitute away from traditional stoves and toward exclusive use of improved stoves.

REACCTING is well-poised to generate useful data on the impact of a cookstove intervention on a wide range of outcomes, from cooking behavior to emissions, exposure, human health, and feedbacks on air quality and regional climate change. A comprehensive and coordinated assessment strategy is being employed to generate consistent and comparable data on all of these outcomes across the four different stove intervention groups. By integrating across these different data streams, REACCTING will allow us to study the impacts of the newly introduced stove technologies from a variety of angles, informing future efforts to combat this pressing public health challenge.

## Abbreviations

BC: Black carbon
CCT: Controlled cooking test
CO: Carbon monoxide
CRP: C-reactive protein
EC: Elemental carbon
FINN: Fire Inventory from NCAR
HDSS: Health and Demographic Surveillance Survey
IL: Interleukin
K-N: Kassena-Nankana
LPG: Liquid petroleum gas
MOx: Metal oxide
NDIR: Non-dispersive infrared
NHRC: Navrongo Health Research Center
NO: Nitrogen monoxide
NO_2_: Nitrogen dioxide
O_3_: Ozone
OC: Organic carbon
PEMS: Portable emission measurement system
PM: Particulate matter
PM_2.5_: Particulate matter less than 2.5 microns in diameter
Pt-Al: Platinum on Alumina
REACCTING: Research on Emissions, Air quality, Climate, and Cooking Technologies in Northern Ghana
RI: Relief International
sCAM: Soluble cell adhesion molecule
SUM: Stove use monitor
TNF-a: Tumor necrosis factor alpha
TSP: Total suspended particulates
TVOCs: Total volatile organic compounds
TZ: Tuo Zaafi (millet-based porridge)
UCB-PATS: University of California at Berkeley Particle And Temperature Sensor
WBT: Water boiling test
WRF-chem: Weather Research Forecasting model with Chemistry
